# The 3-component mixture of power distributions under Bayesian paradigm with application of life span of fatigue fracture

**DOI:** 10.1038/s41598-024-58245-x

**Published:** 2024-04-06

**Authors:** Tahir Abbas, Muhammad Tahir, Muhammad Abid, Samavia Munir, Sajid Ali

**Affiliations:** 1 Present Address: College of Statistical Sciences, University of the Punjab, Lahore, Pakistan; 2 Department of Mathematics, College of Sciences, University of Sharjah, Sharjah, United Arab Emirates; 3 Present Address: Department of Statistics, Government College University, Faisalabad, Pakistan; 4 Department of Statistics, Quaid-i-Azam University, Islamabad, Pakistan

**Keywords:** Bayesian estimation, Power distribution, Prior distribution, Posterior risk, Symmetric and asymmetric loss functions, Censored data, Engineering, Mathematics and computing

## Abstract

Mixture distributions are naturally extra attractive to model the heterogeneous environment of processes in reliability analysis than simple probability models. This focus of the study is to develop and Bayesian inference on the 3-component mixture of power distributions. Under symmetric and asymmetric loss functions, the Bayes estimators and posterior risk using priors are derived. The presentation of Bayes estimators for various sample sizes and test termination time (a fact of time after that test is terminated) is examined in this article. To assess the performance of Bayes estimators in terms of posterior risks, a Monte Carlo simulation along with real data study is presented.

## Introduction

The power distribution is frequently proposed to study the electrical element reliability (Saleem et al.^1^) and in many practical situations, it provides a good fit to data as compared to other distributions, e.g., Rayleigh or gamma distribution. We considered this particular distribution due to skewed nature and applied in different fields like electrical engineering (Amanulla et al.^2^), reliability analysis (Shahzad et al.^3^), city population sizes, stock prices fluctuation, magnitude of earthquakes (Parsa and Murty^4^) and average wealth of a country's citizens etc. However, simple probability distribution may not be well fitted due to heterogeneous environment of reliability data. Therefore, mixture distributions of some suitable distributions are interesting to model the heterogeneous environment of procedures in reliability study. For instance, if the values randomly picked from this population are invented to be considered from three different probability distribution, 3-components mixture of that distribution is recommended. Use of a mixture distribution becomes unavoidable when values are not given for every distribution rather for the overall mixture distribution, so-called direct use of mixture distributions. Li^5^ and Li and Sedransk^6^ discussed type-I mixture distribution (mixture of probability distributions from the same family) and type-II mixture distribution (mixture of probability distributions from various family).

Many researchers have analyzed 2-component mixture models of different probability distributions and applied them to various real life problems under classical and Bayesian framework. Similar to 2-component mixture distribution, some researchers have studied the situations where data are taken from a 3-component mixture distribution. For illustration, in order to know amount of failure because of a definite reason of failure and to expand industrial procedure, Acheson and McElwee^7^ separated electrical tube failures into three types of flaws, namely, gaseous flaws, mechanical flaws, and usual deterioration of the cathode. Davis^8^ also described a mixture data on lifetimes of different parts composed from aircraft failure. Also, Tahir et al.^9^ used the real life mixture data on three parts, namely, Combination of Transformers, Transmitter Tube and Combination of Relays. Haq and Al-Omari^10^ studied the mixture of three Rayleigh distributions using type-I censored data under different scenarios. The application of such methodologies can further be seen in Luo et al.^11^, Wang et al.^12^ and Zhou et al.^13^. Thus, the practical significance of 3-component mixtures of distributions is evident to the cited literature.

Because of time and price restrictions, it is difficult proceed the testing till end value. Consequently, the observations larger than fixed test termination time are retained equally censored observations. It is stating that censoring is an asset of data and it is usually used in real lifetime tests. The practical reason of censoring is stated in Romeu^14^, Gijbels^15^ and Kalbfleisch and Prentice^16^.

Inspired by wide application of mixture distributions, here we define a mixture of the power distributions for capable modeling of practical data under Bayesian paradigm. Different types of loss functions and priors will be assumed to derive Bayes estimators along with posterior risks.

The 3-component mixture of power distributions (3-CMPD) has following pdf and survival function:

1 $$f\left( {y; \, {{\varvec{\Theta}}}} \right) = p_{1} f_{1} \left( y \right) + p_{2} f_{2} \left( y \right) + \left( {1 - p_{1} - p_{2} } \right)f_{3} \left( y \right),\;\;0 < y < 1,$$

2 $$S\left( {y; \, {{\varvec{\Theta}}}} \right) = p_{1} S_{1} \left( y \right) + p_{2} S_{2} \left( y \right) + \left( {1 - p_{1} - p_{2} } \right)S_{3} \left( y \right).$$

where $\lambda_{1} , \, \lambda_{2}$ and $\, \lambda_{3}$ are component parameters, $p_{1}$ and $p_{2}$ are mixing proportions and

3 $${{\varvec{\Theta}}} = \left( {\lambda_{1} , \, \lambda_{2} , \, \lambda_{3} , \, p_{1} , \, p_{2} } \right).$$

The pdf $f_{m} \left( y \right)$ and the survival function $S_{m} \left( y \right)$ of the *m*th component, $m = 1, \, 2, \, 3$, are written as:

4 $$f_{m} \left( y \right) = \lambda_{m} y^{{\lambda_{m} - 1}} \;\;{\text{and}}\;\;S_{m} \left( y \right) = 1 - y^{{\lambda_{m} }} ,\;\; \, \lambda_{m} { > 0}.$$

## Sampling structure for likelihood function

Suppose a data consists of $n$ values from the 3-CMPD are taken in a real life test with fixed $t$ (test termination time). Let $y_{1} , \, y_{2} , \, ...,y_{u}$ be the values that can be observed and remaining $n - u$ greatest values are taken as censored, that is, their failure time cannot be noted. So, ${\varvec{y}}_{1} = y_{11} , \, ..., \, y_{{1u_{1} }}$, ${\varvec{y}}_{2} = y_{21} , \, ..., \, y_{{2u_{2} }}$ and ${\varvec{y}}_{3} = y_{31} , \, ..., \, y_{{3u_{3} }}$ are failed data representing to 1st, 2nd and 3rd subpopulations. Remaining of the data which are greater than $y_{u}$ taken to be censored from each subpopulation, while the numbers $u_{1}$, $u_{2}$ and $u_{3}$ of failed values can be taken from 1st, 2nd and 3rd subpopulations. Rest of the $n_{1} - u_{1}$, $n_{2} - u_{2}$ and $n_{3} - u_{3}$ values are picked as censored data from three subpopulations, whereas $u = u_{1} + u_{2} + u_{3}$. Using the type-I right censored data, ${\mathbf{y}} = \left\{ {\left( {{\varvec{y}}_{1} = y_{11} , \, ..., \, y_{{1u_{1} }} } \right)} \right.$, $\left( {{\varvec{y}}_{2} = y_{21} , \, ..., \, y_{{2u_{2} }} } \right)$, $\left. {\left( {{\varvec{y}}_{3} = y_{31} , \, ..., \, y_{{3u_{3} }} } \right)} \right\}$, the likelihood function is:

5 $$L\left( {{{\varvec{\Theta}}};{\mathbf{y}}} \right) \propto \left\{ {\prod\limits_{w = 1}^{{u_{1} }} {p_{1} f_{1} \left( {y_{1w} } \right)} } \right\}\left\{ {\prod\limits_{w = 1}^{{u_{2} }} {p_{2} f_{2} \left( {y_{2w} } \right)} } \right\}\left\{ {\prod\limits_{w = 1}^{{u_{3} }} {\left( {1 - p_{1} - p_{2} } \right)f_{3} \left( {y_{3w} } \right)} } \right\}\left\{ {S\left( t \right)} \right\}^{n - r} ,$$

On simplification, the likelihood function is:

6 $$\begin{gathered} L\left( {{\mathbf{\Theta ;y}}} \right) \propto \sum\limits_{i = 0}^{n - u} {\sum\limits_{j = 0}^{i} {\sum\limits_{k = 0}^{j} {\left( { - 1} \right)^{i} } \left( \begin{gathered} n - u \hfill \\ \, i \hfill \\ \end{gathered} \right)\left( \begin{gathered} i \hfill \\ j \hfill \\ \end{gathered} \right)\left( \begin{gathered} j \hfill \\ k \hfill \\ \end{gathered} \right)\exp \left\{ { - \lambda_{1} \left( {\left( {i - j} \right)\ln \left( \frac{1}{t} \right) + \sum\limits_{w = 1}^{{u_{1} }} {\ln \left( {\frac{1}{{y_{1w} }}} \right)} } \right)} \right\}} } \hfill \\ \, \times \exp \left\{ { - \lambda_{2} \left( {\left( {j - k} \right)\ln \left( \frac{1}{t} \right) + \sum\limits_{w = 1}^{{u_{2} }} {\ln \left( {\frac{1}{{y_{2w} }}} \right)} } \right)} \right\}\exp \left\{ { - \lambda_{3} \left( {\left( k \right)\ln \left( \frac{1}{t} \right) + \sum\limits_{w = 1}^{{u_{3} }} {\ln \left( {\frac{1}{{y_{3w} }}} \right)} } \right)} \right\} \hfill \\ \, \times \lambda_{1}^{{u_{1} }} \lambda_{2}^{{u_{2} }} \lambda_{3}^{{u_{3} }} p_{1}^{{i - j + u_{1} }} p_{2}^{{j - k + u_{2} }} \left( {1 - p_{1} - p_{2} } \right)^{{k + u_{3} }} \hfill \\ \end{gathered}$$

## Posterior distributions assuming different priors

In this section, using the non-informative priors (NIPs) and informative prior (IP), the posterior distributions of parameters are derived.

### Posterior distribution assuming uniform prior (UP)

If no prior or additional prior knowledge is given, the use of UP and JP (Jeffreys’ prior) as NIPs are recommended in Bayesian estimation. We assume the ${\text{uniform (}}0, \, \infty {)}$ for component parameter $\lambda_{m} \, (m = 1, \, 2, \, 3)$ and the ${\text{uniform (}}0,{ 1)}$ for the proportion parameter $p_{s} \, (s = 1, \, 2)$. The joint prior distribution is $\pi_{1} \left( {{\varvec{\Theta}}} \right) \propto 1$. Thus, the joint posterior distribution given censored data ${\mathbf{y}}$ is:

7 $$q_{1} \left( {{{\varvec{\Theta}}}\left| {\mathbf{y}} \right.} \right) = \frac{{L\left( {{\mathbf{\Theta ;y}}} \right)\pi_{1} \left( {{\varvec{\Theta}}} \right)}}{{\int\limits_{{{\varvec{\Psi}}}} {L\left( {{\mathbf{\Theta ;y}}} \right)\pi_{1} \left( {{\varvec{\Theta}}} \right)d{{\varvec{\Theta}}}} }}.$$

8 $$q_{1} \left( {{{\varvec{\Theta}}}\left| {\mathbf{y}} \right.} \right) = \frac{{\sum\limits_{i = 0}^{n - u} {\sum\limits_{j = 0}^{i} {\sum\limits_{k = 0}^{j} {\left( { - 1} \right)^{i} } \left( \begin{gathered} n - u \hfill \\ \, i \hfill \\ \end{gathered} \right)\left( \begin{gathered} i \hfill \\ j \hfill \\ \end{gathered} \right)\left( \begin{gathered} j \hfill \\ k \hfill \\ \end{gathered} \right)\exp \left( { - B_{11} \lambda_{1} } \right)\exp \left( { - B_{21} \lambda_{2} } \right)} } \exp \left( { - B_{31} \lambda_{3} } \right)p_{1}^{{A_{01} - 1}} p_{2}^{{B_{01} - 1}} \left( {1 - p_{1} - p_{2} } \right)^{{C_{01} - 1}} }}{{\Omega_{1} \, \lambda_{1}^{{1 - A_{11} }} \lambda_{2}^{{1 - A_{21} }} \lambda_{3}^{{1 - A_{31} }} }},$$

where $$A_{11} = 1 + u_{1}$$, $$A_{21} = 1 + u_{2}$$, $$A_{31} = 1 + u_{3}$$, $$B_{11} = \left( {i - j} \right)\ln \left( \frac{1}{t} \right) + \sum\limits_{w = 1}^{{u_{1} }} {\ln \left( {\frac{1}{{y_{1w} }}} \right)}$$, $$B_{21} = \left( {j - k} \right)\ln \left( \frac{1}{t} \right) + \sum\limits_{w = 1}^{{u_{2} }} {\ln \left( {\frac{1}{{y_{2w} }}} \right)}$$, $$B_{31} = \left( k \right)\ln \left( \frac{1}{t} \right) + \sum\limits_{w = 1}^{{u_{3} }} {\ln \left( {\frac{1}{{y_{3w} }}} \right)}$$, $$A_{01} = i + u_{1} + 1 - j$$, $$B_{01} = j + u_{2} + 1 - k$$, $$C_{01} = k + u_{3} + 1$$,$$\Omega_{1} = \sum\limits_{i = 0}^{n - u} {\sum\limits_{j = 0}^{i} {\sum\limits_{k = 0}^{j} {\left( { - 1} \right)^{i} } \left( \begin{gathered} n - u \hfill \\ \, i \hfill \\ \end{gathered} \right)\left( \begin{gathered} i \hfill \\ j \hfill \\ \end{gathered} \right)\left( \begin{gathered} j \hfill \\ k \hfill \\ \end{gathered} \right)\Gamma \left( {A_{11} } \right)\Gamma \left( {A_{21} } \right)\Gamma \left( {A_{31} } \right)B_{11}^{{ - A_{11} }} B_{21}^{{ - A_{21} }} B_{31}^{{ - A_{31} }} } } B\left( {A_{01} ,B_{01} ,C_{01} } \right)$$.

After simplification, the marginal posterior distributions are derived as:

9 $$g_{1} \left( {\lambda_{1} \left| {\mathbf{y}} \right.} \right) = \frac{1}{{\Omega_{1} }}\sum\limits_{i = 0}^{n - u} {\sum\limits_{j = 0}^{i} {\sum\limits_{k = 0}^{j} {\left( { - 1} \right)^{i} } \left( \begin{gathered} n - u \hfill \\ \, i \hfill \\ \end{gathered} \right)\left( \begin{gathered} i \hfill \\ j \hfill \\ \end{gathered} \right)\left( \begin{gathered} j \hfill \\ k \hfill \\ \end{gathered} \right)B\left( {A_{01} ,C_{01} } \right)B\left( {B_{01} ,A_{01} + C_{01} } \right)\Gamma \left( {A_{21} } \right)\Gamma \left( {A_{31} } \right)B_{21}^{{ - A_{21} }} B_{31}^{{ - A_{31} }} } } \lambda_{1}^{{A_{11} - 1}} \exp \left( { - B_{11} \lambda_{1} } \right)$$

10 $$g_{1} \left( {\lambda_{2} \left| {\mathbf{y}} \right.} \right) = \frac{1}{{\Omega_{1} }}\sum\limits_{i = 0}^{n - u} {\sum\limits_{j = 0}^{i} {\sum\limits_{k = 0}^{j} {\left( { - 1} \right)^{i} } \left( \begin{gathered} n - u \hfill \\ \, i \hfill \\ \end{gathered} \right)\left( \begin{gathered} i \hfill \\ j \hfill \\ \end{gathered} \right)\left( \begin{gathered} j \hfill \\ k \hfill \\ \end{gathered} \right)B\left( {A_{01} ,C_{01} } \right)B\left( {B_{01} ,A_{01} + C_{01} } \right)\Gamma \left( {A_{11} } \right)\Gamma \left( {A_{31} } \right)B_{11}^{{ - A_{11} }} B_{31}^{{ - A_{31} }} } } \lambda_{2}^{{A_{21} - 1}} \exp \left( { - B_{21} \lambda_{2} } \right)$$

11 $$g_{1} \left( {\lambda_{3} \left| {\mathbf{y}} \right.} \right) = \frac{1}{{\Omega_{1} }}\sum\limits_{i = 0}^{n - u} {\sum\limits_{j = 0}^{i} {\sum\limits_{k = 0}^{j} {\left( { - 1} \right)^{i} } \left( \begin{gathered} n - u \hfill \\ \, i \hfill \\ \end{gathered} \right)\left( \begin{gathered} i \hfill \\ j \hfill \\ \end{gathered} \right)\left( \begin{gathered} j \hfill \\ k \hfill \\ \end{gathered} \right)B\left( {A_{01} ,C_{01} } \right)B\left( {B_{01} ,A_{01} + C_{01} } \right)\Gamma \left( {A_{21} } \right)\Gamma \left( {A_{31} } \right)B_{11}^{{ - A_{11} }} B_{21}^{{ - A_{21} }} } } \lambda_{3}^{{A_{31} - 1}} \exp \left( { - B_{31} \lambda_{3} } \right)$$

12 $$g_{1} \left( {p_{1} \left| {\mathbf{y}} \right.} \right) = \frac{1}{{\Omega_{1} }}\sum\limits_{i = 0}^{n - u} {\sum\limits_{j = 0}^{i} {\sum\limits_{k = 0}^{j} {\left( { - 1} \right)^{i} } \left( \begin{gathered} n - u \hfill \\ \, i \hfill \\ \end{gathered} \right)\left( \begin{gathered} i \hfill \\ j \hfill \\ \end{gathered} \right)\left( \begin{gathered} j \hfill \\ k \hfill \\ \end{gathered} \right)\Gamma \left( {A_{11} } \right)\Gamma \left( {A_{21} } \right)\Gamma \left( {A_{31} } \right)B_{11}^{{ - A_{11} }} B_{21}^{{ - A_{21} }} B_{31}^{{ - A_{31} }} } } B\left( {B_{01} ,C_{01} } \right)p_{1}^{{A_{01} - 1}} \left( {1 - p_{1} } \right)^{{B_{01} + C_{01} - 1}}$$

13 $$g_{1} \left( {p_{2} \left| {\mathbf{y}} \right.} \right) = \frac{1}{{\Omega_{1} }}\sum\limits_{i = 0}^{n - u} {\sum\limits_{j = 0}^{i} {\sum\limits_{k = 0}^{j} {\left( { - 1} \right)^{i} } \left( \begin{gathered} n - u \hfill \\ \, i \hfill \\ \end{gathered} \right)\left( \begin{gathered} i \hfill \\ j \hfill \\ \end{gathered} \right)\left( \begin{gathered} j \hfill \\ k \hfill \\ \end{gathered} \right)\Gamma \left( {A_{11} } \right)\Gamma \left( {A_{21} } \right)\Gamma \left( {A_{31} } \right)B_{11}^{{ - A_{11} }} B_{21}^{{ - A_{21} }} B_{31}^{{ - A_{31} }} } } B\left( {A_{01} ,C_{01} } \right)p_{2}^{{B_{01} - 1}} \left( {1 - p_{2} } \right)^{{A_{01} + C_{01} - 1}} .$$

### Posterior distribution assuming Jeffreys’ prior (JP)

Jeffreys^17,18^ suggested a formula for finding the JP as: $p\left( {\lambda_{m} } \right) \propto \left[ {\left| { - E\left\{ {\frac{{\partial^{2} \ln L\left( {\lambda_{m} \left| {{\varvec{y}}_{m} } \right.} \right)}}{{\partial \lambda_{m}^{2} }}} \right\}} \right|} \right]^{{{1 \mathord{\left/ {\vphantom {1 2}} \right. \kern-0pt} 2}}}$, where $- E\left\{ {\frac{{\partial^{2} \ln L\left( {\lambda_{m} \left| {{\varvec{y}}_{m} } \right.} \right)}}{{\partial \lambda_{m}^{2} }}} \right\}$ is Fisher’s information. Here, we take prior distributions of $p_{s} \, (s = 1, \, 2)$ are ${\text{uniform (}}0,{ 1)}$. So, the joint posterior distribution given censored data ${\mathbf{y}}$ using $\pi_{2} \left( {{\varvec{\Theta}}} \right) \propto \frac{1}{{\lambda_{1} \lambda_{2} \lambda_{3} }}$ as joint prior distribution is:

14 $$q_{2} \left( {{{\varvec{\Theta}}}\left| {\mathbf{y}} \right.} \right) = \frac{{\sum\limits_{i = 0}^{n - u} {\sum\limits_{j = 0}^{i} {\sum\limits_{k = 0}^{j} {\left( { - 1} \right)^{i} } \left( \begin{gathered} n - u \hfill \\ \, i \hfill \\ \end{gathered} \right)\left( \begin{gathered} i \hfill \\ j \hfill \\ \end{gathered} \right)\left( \begin{gathered} j \hfill \\ k \hfill \\ \end{gathered} \right)\exp \left( { - B_{12} \lambda_{1} } \right)\exp \left( { - B_{22} \lambda_{2} } \right)} } \exp \left( { - B_{32} \lambda_{3} } \right)p_{1}^{{A_{02} - 1}} p_{2}^{{B_{02} - 1}} \left( {1 - p_{1} - p_{2} } \right)^{{C_{02} - 1}} }}{{\Omega_{2} \, \lambda_{1}^{{1 - A_{12} }} \lambda_{2}^{{1 - A_{22} }} \lambda_{3}^{{1 - A_{32} }} }},$$

where $$A_{12} = u_{1}$$, $$A_{22} = u_{2}$$, $$A_{32} = u_{3}$$, $$B_{12} = \left( {i - j} \right)\ln \left( \frac{1}{t} \right) + \sum\limits_{w = 1}^{{u_{1} }} {\ln \left( {\frac{1}{{y_{1w} }}} \right)}$$, $$B_{22} = \left( {j - k} \right)\ln \left( \frac{1}{t} \right) + \sum\limits_{w = 1}^{{u_{2} }} {\ln \left( {\frac{1}{{y_{2w} }}} \right)}$$, $$B_{32} = \left( k \right)\ln \left( \frac{1}{t} \right) + \sum\limits_{w = 1}^{{u_{3} }} {\ln \left( {\frac{1}{{y_{3w} }}} \right)}$$, $$A_{02} = i - j + u_{1} + 1$$, $$B_{02} = j - k + u_{2} + 1$$, $$C_{02} = k + u_{3} + 1$$, $$\Omega_{2} = \sum\limits_{i = 0}^{n - u} {\sum\limits_{j = 0}^{i} {\sum\limits_{k = 0}^{j} {\left( { - 1} \right)^{i} } \left( \begin{gathered} n - u \hfill \\ \, i \hfill \\ \end{gathered} \right)\left( \begin{gathered} i \hfill \\ j \hfill \\ \end{gathered} \right)\left( \begin{gathered} j \hfill \\ k \hfill \\ \end{gathered} \right)B_{12}^{{ - A_{12} }} B_{22}^{{ - A_{22} }} B_{32}^{{ - A_{32} }} \Gamma \left( {A_{12} } \right)\Gamma \left( {A_{22} } \right)\Gamma \left( {A_{32} } \right)B\left( {A_{02} ,B_{02} ,C_{02} } \right)} }$$.

The marginal posterior distributions are derived as:

15 $$g_{2} \left( {\lambda_{1} \left| {\mathbf{y}} \right.} \right) = \frac{1}{{\Omega_{2} }}\sum\limits_{i = 0}^{n - u} {\sum\limits_{j = 0}^{i} {\sum\limits_{k = 0}^{j} {\left( { - 1} \right)^{i} } \left( \begin{gathered} n - u \hfill \\ \, i \hfill \\ \end{gathered} \right)\left( \begin{gathered} i \hfill \\ j \hfill \\ \end{gathered} \right)\left( \begin{gathered} j \hfill \\ k \hfill \\ \end{gathered} \right)B\left( {A_{02} ,C_{02} } \right)B\left( {B_{02} ,A_{02} + C_{02} } \right)\Gamma \left( {A_{22} } \right)\Gamma \left( {A_{32} } \right)B_{22}^{{ - A_{22} }} B_{32}^{{ - A_{32} }} } } \lambda_{1}^{{A_{12} - 1}} \exp \left( { - B_{12} \lambda_{1} } \right)$$

16 $$g_{2} \left( {\lambda_{2} \left| {\mathbf{y}} \right.} \right) = \frac{1}{{\Omega_{2} }}\sum\limits_{i = 0}^{n - u} {\sum\limits_{j = 0}^{i} {\sum\limits_{k = 0}^{j} {\left( { - 1} \right)^{i} } \left( \begin{gathered} n - u \hfill \\ \, i \hfill \\ \end{gathered} \right)\left( \begin{gathered} i \hfill \\ j \hfill \\ \end{gathered} \right)\left( \begin{gathered} j \hfill \\ k \hfill \\ \end{gathered} \right)B\left( {A_{02} ,C_{02} } \right)B\left( {B_{02} ,A_{02} + C_{02} } \right)\Gamma \left( {A_{12} } \right)\Gamma \left( {A_{32} } \right)B_{12}^{{ - A_{12} }} B_{32}^{{ - A_{32} }} } } \lambda_{2}^{{A_{22} - 1}} \exp \left( { - B_{22} \lambda_{2} } \right)$$

17 $$g_{2} \left( {\lambda_{3} \left| {\mathbf{y}} \right.} \right) = \frac{1}{{\Omega_{2} }}\sum\limits_{i = 0}^{n - u} {\sum\limits_{j = 0}^{i} {\sum\limits_{k = 0}^{j} {\left( { - 1} \right)^{i} } \left( \begin{gathered} n - u \hfill \\ \, i \hfill \\ \end{gathered} \right)\left( \begin{gathered} i \hfill \\ j \hfill \\ \end{gathered} \right)\left( \begin{gathered} j \hfill \\ k \hfill \\ \end{gathered} \right)B\left( {A_{02} ,C_{02} } \right)B\left( {B_{02} ,A_{02} + C_{02} } \right)\Gamma \left( {A_{22} } \right)\Gamma \left( {A_{32} } \right)B_{12}^{{ - A_{12} }} B_{22}^{{ - A_{22} }} } } \lambda_{3}^{{A_{32} - 1}} \exp \left( { - B_{32} \lambda_{3} } \right)$$

18 $$g_{2} \left( {p_{1} \left| {\mathbf{y}} \right.} \right) = \frac{1}{{\Omega_{2} }}\sum\limits_{i = 0}^{n - u} {\sum\limits_{j = 0}^{i} {\sum\limits_{k = 0}^{j} {\left( { - 1} \right)^{i} } \left( \begin{gathered} n - u \hfill \\ \, i \hfill \\ \end{gathered} \right)\left( \begin{gathered} i \hfill \\ j \hfill \\ \end{gathered} \right)\left( \begin{gathered} j \hfill \\ k \hfill \\ \end{gathered} \right)\Gamma \left( {A_{12} } \right)\Gamma \left( {A_{22} } \right)\Gamma \left( {A_{32} } \right)B_{12}^{{ - A_{12} }} B_{22}^{{ - A_{22} }} B_{32}^{{ - A_{32} }} } } B\left( {B_{02} ,C_{02} } \right)p_{1}^{{A_{02} - 1}} \left( {1 - p_{1} } \right)^{{B_{02} + C_{02} - 1}}$$

19 $$g_{2} \left( {p_{2} \left| {\mathbf{y}} \right.} \right) = \frac{1}{{\Omega_{2} }}\sum\limits_{i = 0}^{n - u} {\sum\limits_{j = 0}^{i} {\sum\limits_{k = 0}^{j} {\left( { - 1} \right)^{i} } \left( \begin{gathered} n - u \hfill \\ \, i \hfill \\ \end{gathered} \right)\left( \begin{gathered} i \hfill \\ j \hfill \\ \end{gathered} \right)\left( \begin{gathered} j \hfill \\ k \hfill \\ \end{gathered} \right)\Gamma \left( {A_{12} } \right)\Gamma \left( {A_{22} } \right)\Gamma \left( {A_{32} } \right)B_{12}^{{ - A_{12} }} B_{22}^{{ - A_{22} }} B_{32}^{{ - A_{32} }} } } B\left( {A_{02} ,C_{02} } \right)p_{2}^{{B_{02} - 1}} \left( {1 - p_{2} } \right)^{{A_{02} + C_{02} - 1}} .$$

### Posterior distribution assuming the Informative prior

As an IP, we assume $Gamma\left( {a_{m} , \, b_{m} } \right)$ for parameter $\lambda_{m}$ and $Bivariate \, Beta\left( {a, \, b, \, c} \right)$ for the proportion parameter $p_{s}$. The joint prior distribution is:

20 $$\pi_{3} \left( {{\varvec{\Theta}}} \right) = \frac{{b_{1}^{{a_{1} }} }}{{\Gamma \left( {a_{1} } \right)}}\frac{{b_{2}^{{a_{2} }} }}{{\Gamma \left( {a_{2} } \right)}}\frac{{b_{3}^{{a_{3} }} }}{{\Gamma \left( {a_{3} } \right)}}\frac{1}{{B\left( {a,b,c} \right)}}\frac{{e^{{ - b_{1} \lambda_{1} }} }}{{\lambda_{1}^{{1 - a_{1} }} }}\frac{{e^{{ - b_{2} \lambda_{2} }} }}{{\lambda_{2}^{{1 - a_{2} }} }}\frac{{e^{{ - b_{3} \lambda_{3} }} }}{{\lambda_{3}^{{1 - a_{3} }} }}p_{1}^{a - 1} p_{2}^{b - 1} \left( {1 - p_{1} - p_{2} } \right)^{c - 1} .$$

So, the joint posterior distribution given censored data ${\mathbf{y}}$ is:

21 $$q_{3} \left( {{{\varvec{\Theta}}}\left| {\mathbf{y}} \right.} \right) = \frac{{\sum\limits_{i = 0}^{n - u} {\sum\limits_{j = 0}^{i} {\sum\limits_{k = 0}^{j} {\left( { - 1} \right)^{i} } \left( \begin{gathered} n - u \hfill \\ \, i \hfill \\ \end{gathered} \right)\left( \begin{gathered} i \hfill \\ j \hfill \\ \end{gathered} \right)\left( \begin{gathered} j \hfill \\ k \hfill \\ \end{gathered} \right)\exp \left( { - B_{13} \lambda_{1} } \right)\exp \left( { - B_{23} \lambda_{2} } \right)} } \exp \left( { - B_{33} \lambda_{3} } \right)p_{1}^{{A_{03} - 1}} p_{2}^{{B_{03} - 1}} \left( {1 - p_{1} - p_{2} } \right)^{{C_{03} - 1}} }}{{\Omega_{3} \, \lambda_{1}^{{1 - A_{13} }} \lambda_{2}^{{1 - A_{23} }} \lambda_{3}^{{1 - A_{33} }} }},$$

where $$A_{13} = a_{1} + u_{1}$$, $$B_{13} = \sum\limits_{w = 1}^{{u_{1} }} {\ln \left( {\frac{1}{{y_{1w} }}} \right)} + \left( {i - j} \right)\ln \left( \frac{1}{t} \right) + b_{1}$$, $$A_{23} = a_{2} + u_{2}$$, $$B_{23} = \sum\limits_{w = 1}^{{u_{2} }} {\ln \left( {\frac{1}{{y_{2w} }}} \right)} + \left( {j - k} \right)\ln \left( \frac{1}{t} \right) + b_{2}$$, $$A_{33} = a_{3} + u_{3}$$, $$B_{33} = \sum\limits_{w = 1}^{{u_{3} }} {\ln \left( {\frac{1}{{y_{3w} }}} \right)} + \left( k \right)\ln \left( \frac{1}{t} \right) + b_{3}$$, $$A_{03} = i - j + u_{1} + a$$, $$B_{03} = j - k + u_{2} + b$$, $$C_{03} = k + u_{3} + c$$,$$\Omega_{3} = \sum\limits_{i = 0}^{n - u} {\sum\limits_{j = 0}^{i} {\sum\limits_{k = 0}^{j} {\left( { - 1} \right)^{i} } \left( \begin{gathered} n - u \hfill \\ \, i \hfill \\ \end{gathered} \right)\left( \begin{gathered} i \hfill \\ j \hfill \\ \end{gathered} \right)\left( \begin{gathered} j \hfill \\ k \hfill \\ \end{gathered} \right)B\left( {A_{03} ,B_{03} ,C_{03} } \right)\Gamma \left( {A_{13} } \right)\Gamma \left( {A_{23} } \right)\Gamma \left( {A_{33} } \right)B_{13}^{{ - A_{13} }} B_{23}^{{ - A_{23} }} B_{33}^{{ - A_{33} }} } }$$.

The marginal posterior distributions are derived as:

22 $$g_{3} \left( {\lambda_{1} \left| {\mathbf{y}} \right.} \right) = \frac{1}{{\Omega_{3} }}\sum\limits_{i = 0}^{n - u} {\sum\limits_{j = 0}^{i} {\sum\limits_{k = 0}^{j} {\left( { - 1} \right)^{i} } \left( \begin{gathered} n - u \hfill \\ \, i \hfill \\ \end{gathered} \right)\left( \begin{gathered} i \hfill \\ j \hfill \\ \end{gathered} \right)\left( \begin{gathered} j \hfill \\ k \hfill \\ \end{gathered} \right)B\left( {A_{03} ,C_{03} } \right)B\left( {B_{03} ,A_{03} + C_{03} } \right)\Gamma \left( {A_{23} } \right)\Gamma \left( {A_{33} } \right)B_{23}^{{ - A_{23} }} B_{33}^{{ - A_{33} }} } } \lambda_{1}^{{A_{13} - 1}} \exp \left( { - B_{13} \lambda_{1} } \right)$$

23 $$g_{3} \left( {\lambda_{2} \left| {\mathbf{y}} \right.} \right) = \frac{1}{{\Omega_{3} }}\sum\limits_{i = 0}^{n - u} {\sum\limits_{j = 0}^{i} {\sum\limits_{k = 0}^{j} {\left( { - 1} \right)^{i} } \left( \begin{gathered} n - u \hfill \\ \, i \hfill \\ \end{gathered} \right)\left( \begin{gathered} i \hfill \\ j \hfill \\ \end{gathered} \right)\left( \begin{gathered} j \hfill \\ k \hfill \\ \end{gathered} \right)B\left( {A_{03} ,C_{03} } \right)B\left( {B_{03} ,A_{03} + C_{03} } \right)\Gamma \left( {A_{13} } \right)\Gamma \left( {A_{33} } \right)B_{13}^{{ - A_{13} }} B_{33}^{{ - A_{33} }} } } \lambda_{2}^{{A_{23} - 1}} \exp \left( { - B_{23} \lambda_{2} } \right)$$

24 $$g_{3} \left( {\lambda_{3} \left| {\mathbf{y}} \right.} \right) = \frac{1}{{\Omega_{3} }}\sum\limits_{i = 0}^{n - u} {\sum\limits_{j = 0}^{i} {\sum\limits_{k = 0}^{j} {\left( { - 1} \right)^{i} } \left( \begin{gathered} n - u \hfill \\ \, i \hfill \\ \end{gathered} \right)\left( \begin{gathered} i \hfill \\ j \hfill \\ \end{gathered} \right)\left( \begin{gathered} j \hfill \\ k \hfill \\ \end{gathered} \right)B\left( {A_{03} ,C_{03} } \right)B\left( {B_{03} ,A_{03} + C_{03} } \right)\Gamma \left( {A_{23} } \right)\Gamma \left( {A_{33} } \right)B_{13}^{{ - A_{13} }} B_{23}^{{ - A_{23} }} } } \lambda_{3}^{{A_{33} - 1}} \exp \left( { - B_{33} \lambda_{3} } \right)$$

25 $$g_{3} \left( {p_{1} \left| {\mathbf{y}} \right.} \right) = \frac{1}{{\Omega_{3} }}\sum\limits_{i = 0}^{n - u} {\sum\limits_{j = 0}^{i} {\sum\limits_{k = 0}^{j} {\left( { - 1} \right)^{i} } \left( \begin{gathered} n - u \hfill \\ \, i \hfill \\ \end{gathered} \right)\left( \begin{gathered} i \hfill \\ j \hfill \\ \end{gathered} \right)\left( \begin{gathered} j \hfill \\ k \hfill \\ \end{gathered} \right)\Gamma \left( {A_{13} } \right)\Gamma \left( {A_{23} } \right)\Gamma \left( {A_{33} } \right)B_{13}^{{ - A_{13} }} B_{23}^{{ - A_{23} }} B_{33}^{{ - A_{33} }} } } B\left( {B_{03} ,C_{03} } \right)p_{1}^{{A_{03} - 1}} \left( {1 - p_{1} } \right)^{{B_{03} + C_{03} - 1}}$$

26 $$g_{3} \left( {p_{2} \left| {\mathbf{y}} \right.} \right) = \frac{1}{{\Omega_{3} }}\sum\limits_{i = 0}^{n - u} {\sum\limits_{j = 0}^{i} {\sum\limits_{k = 0}^{j} {\left( { - 1} \right)^{i} } \left( \begin{gathered} n - u \hfill \\ \, i \hfill \\ \end{gathered} \right)\left( \begin{gathered} i \hfill \\ j \hfill \\ \end{gathered} \right)\left( \begin{gathered} j \hfill \\ k \hfill \\ \end{gathered} \right)\Gamma \left( {A_{13} } \right)\Gamma \left( {A_{23} } \right)\Gamma \left( {A_{33} } \right)B_{13}^{{ - A_{13} }} B_{23}^{{ - A_{23} }} B_{33}^{{ - A_{33} }} } } B\left( {A_{03} ,C_{03} } \right)p_{2}^{{B_{03} - 1}} \left( {1 - p_{2} } \right)^{{A_{03} + C_{03} - 1}} .$$

## Bayesian estimation using loss functions

Here, we derived the Bayes estimators (BEs) and their respective posterior risks (PRs) using Squared error loss function (SELF) and quadratic loss function (QLF) as symmetric loss functions, whereas, DeGroot loss function (DLF) and precautionary loss function (PLF) as asymmetric loss functions. The SELF, PLF and DLF introduced by Legendre^19^, Norstrom^20^ and DeGroot^21^, respectively. For a given posterior, the general expressions of the BEs and PRs are presented in Table 1.

**Table 1:** The BEs and PRs under loss functions.

Loss function $= L\left( {\lambda , \, \omega } \right)$	BE $= \hat{\omega }$	PR $= \rho \left( {\hat{\omega }} \right)$
$SELF = \left( {\lambda \user2{ - }\omega } \right)^{2}$	$E\left( \lambda \right)$	$Var\left( \lambda \right)$
$QLF = \frac{{\left( {\lambda \user2{ - }\omega } \right)^{2} }}{\lambda }$	$\frac{{E\left( {\lambda^{ - 1} } \right)}}{{E\left( {\lambda^{ - 2} } \right)}}$	$1 - \frac{{E\left( {\lambda^{ - 1} } \right)}}{{E\left( {\lambda^{ - 2} } \right)}}$
$PLF = \frac{{\left( {\lambda \user2{ - }\omega } \right)^{2} }}{\omega }$	$\left\{ {E\left( {\lambda^{2} } \right)} \right\}^{{{1 \mathord{\left/ {\vphantom {1 2}} \right. \kern-0pt} 2}}}$	$2\sqrt {E\left( {\lambda^{2} } \right)} - 2E\left( \lambda \right)$
$DLF = \left( {\frac{{\lambda \user2{ - }\omega }}{\omega }} \right)^{2}$	$\frac{{E\left( {\lambda^{2} } \right)}}{E\left( \lambda \right)}$	$\frac{Var\left( \lambda \right)}{{E\left( {\lambda^{2} } \right)}}$

### Expressions for BEs and PRs using SELF

After simplification, the closed form expressions of BEs and PRs are given below:

27 $$\hat{\lambda }_{1} = \frac{1}{{\Omega_{v} }}\sum\limits_{i = 0}^{n - u} {\sum\limits_{j = 0}^{i} {\sum\limits_{k = 0}^{j} {\left( { - 1} \right)^{i} } \left( \begin{gathered} n - u \hfill \\ \, i \hfill \\ \end{gathered} \right)\left( \begin{gathered} i \hfill \\ j \hfill \\ \end{gathered} \right)\left( \begin{gathered} j \hfill \\ k \hfill \\ \end{gathered} \right)B\left( {A_{0v} ,C_{0v} } \right)B\left( {B_{0v} ,A_{0v} + C_{0v} } \right)B_{1v}^{{ - \left( {A_{1v} + 1} \right)}} \Gamma \left( {A_{1v} + 1} \right)B_{2v}^{{ - A_{2v} }} \Gamma \left( {A_{2v} } \right)B_{3v}^{{ - A_{3v} }} \Gamma \left( {A_{3v} } \right)} }$$

28 $$\hat{\lambda }_{2} = \frac{1}{{\Omega_{v} }}\sum\limits_{i = 0}^{n - u} {\sum\limits_{j = 0}^{i} {\sum\limits_{k = 0}^{j} {\left( { - 1} \right)^{i} } \left( \begin{gathered} n - u \hfill \\ \, i \hfill \\ \end{gathered} \right)\left( \begin{gathered} i \hfill \\ j \hfill \\ \end{gathered} \right)\left( \begin{gathered} j \hfill \\ k \hfill \\ \end{gathered} \right)B\left( {A_{0v} ,C_{0v} } \right)B\left( {B_{0v} ,A_{0v} + C_{0v} } \right)B_{1v}^{{ - A_{1v} }} \Gamma \left( {A_{1v} } \right)B_{2v}^{{ - \left( {A_{2v} + 1} \right)}} \Gamma \left( {A_{2v} + 1} \right)B_{3v}^{{ - A_{3v} }} \Gamma \left( {A_{3v} } \right)} }$$

29 $$\hat{\lambda }_{3} = \frac{1}{{\Omega_{v} }}\sum\limits_{i = 0}^{n - u} {\sum\limits_{j = 0}^{i} {\sum\limits_{k = 0}^{j} {\left( { - 1} \right)^{i} } \left( \begin{gathered} n - u \hfill \\ \, i \hfill \\ \end{gathered} \right)\left( \begin{gathered} i \hfill \\ j \hfill \\ \end{gathered} \right)\left( \begin{gathered} j \hfill \\ k \hfill \\ \end{gathered} \right)B\left( {A_{0v} ,C_{0v} } \right)B\left( {B_{0v} ,A_{0v} + C_{0v} } \right)B_{1v}^{{ - A_{1v} }} \Gamma \left( {A_{1v} } \right)B_{2v}^{{ - A_{2v} }} \Gamma \left( {A_{2v} } \right)B_{3v}^{{ - \left( {A_{3v} + 1} \right)}} \Gamma \left( {A_{3v} + 1} \right)} }$$

30 $$\hat{p}_{1} = \frac{1}{{\Omega_{v} }}\sum\limits_{i = 0}^{n - u} {\sum\limits_{j = 0}^{i} {\sum\limits_{k = 0}^{j} {\left( { - 1} \right)^{i} } \left( \begin{gathered} n - u \hfill \\ \, i \hfill \\ \end{gathered} \right)\left( \begin{gathered} i \hfill \\ j \hfill \\ \end{gathered} \right)\left( \begin{gathered} j \hfill \\ k \hfill \\ \end{gathered} \right)B\left( {B_{0v} ,C_{0v} } \right)B\left( {A_{0v} + 1,B_{0v} + C_{0v} } \right)B_{1v}^{{ - A_{1v} }} \Gamma \left( {A_{1v} } \right)B_{2v}^{{ - A_{2v} }} \Gamma \left( {A_{2v} } \right)B_{3v}^{{ - A_{3v} }} \Gamma \left( {A_{3v} } \right)} }$$

31 $$\hat{p}_{2} = \frac{1}{{\Omega_{v} }}\sum\limits_{i = 0}^{n - u} {\sum\limits_{j = 0}^{i} {\sum\limits_{k = 0}^{j} {\left( { - 1} \right)^{i} } \left( \begin{gathered} n - u \hfill \\ \, i \hfill \\ \end{gathered} \right)\left( \begin{gathered} i \hfill \\ j \hfill \\ \end{gathered} \right)\left( \begin{gathered} j \hfill \\ k \hfill \\ \end{gathered} \right)B\left( {A_{0v} ,C_{0v} } \right)B\left( {B_{0v} + 1,A_{0v} + C_{0v} } \right)B_{1v}^{{ - A_{1v} }} \Gamma \left( {A_{1v} } \right)B_{2v}^{{ - A_{2v} }} \Gamma \left( {A_{2v} } \right)B_{3v}^{{ - A_{3v} }} \Gamma \left( {A_{3v} } \right)} }$$

32 $$\rho \left( {\hat{\lambda }_{1} } \right) = \frac{1}{{\Omega_{v} }}\sum\limits_{i = 0}^{n - u} {\sum\limits_{j = 0}^{i} {\sum\limits_{k = 0}^{j} {\left( { - 1} \right)^{i} } \left( \begin{gathered} n - u \hfill \\ \, i \hfill \\ \end{gathered} \right)\left( \begin{gathered} i \hfill \\ j \hfill \\ \end{gathered} \right)\left( \begin{gathered} j \hfill \\ k \hfill \\ \end{gathered} \right)B\left( {A_{0v} ,C_{0v} } \right)B\left( {B_{0v} ,A_{0v} + C_{0v} } \right)B_{1v}^{{ - \left( {A_{1v} + 2} \right)}} \Gamma \left( {A_{1v} + 2} \right)B_{2v}^{{ - A_{2v} }} \Gamma \left( {A_{2v} } \right)B_{3v}^{{ - A_{3v} }} \Gamma \left( {A_{3v} } \right)} } - \left\{ {\hat{\lambda }_{1} } \right\}^{2}$$

33 $$\rho \left( {\hat{\lambda }_{2} } \right) = \frac{1}{{\Omega_{v} }}\sum\limits_{i = 0}^{n - u} {\sum\limits_{j = 0}^{i} {\sum\limits_{k = 0}^{j} {\left( { - 1} \right)^{i} } \left( \begin{gathered} n - u \hfill \\ \, i \hfill \\ \end{gathered} \right)\left( \begin{gathered} i \hfill \\ j \hfill \\ \end{gathered} \right)\left( \begin{gathered} j \hfill \\ k \hfill \\ \end{gathered} \right)B\left( {A_{0v} ,C_{0v} } \right)B\left( {B_{0v} ,A_{0v} + C_{0v} } \right)B_{1v}^{{ - A_{1v} }} \Gamma \left( {A_{1v} } \right)B_{2v}^{{ - \left( {A_{2v} + 2} \right)}} \Gamma \left( {A_{2v} + 2} \right)B_{3v}^{{ - A_{3v} }} \Gamma \left( {A_{3v} } \right)} } - \left\{ {\hat{\lambda }_{2} } \right\}^{2}$$

34 $$\rho \left( {\hat{\lambda }_{3} } \right) = \frac{1}{{\Omega_{v} }}\sum\limits_{i = 0}^{n - u} {\sum\limits_{j = 0}^{i} {\sum\limits_{k = 0}^{j} {\left( { - 1} \right)^{i} } \left( \begin{gathered} n - u \hfill \\ \, i \hfill \\ \end{gathered} \right)\left( \begin{gathered} i \hfill \\ j \hfill \\ \end{gathered} \right)\left( \begin{gathered} j \hfill \\ k \hfill \\ \end{gathered} \right)B\left( {A_{0v} ,C_{0v} } \right)B\left( {B_{0v} ,A_{0v} + C_{0v} } \right)B_{1v}^{{ - A_{1v} }} \Gamma \left( {A_{1v} } \right)B_{2v}^{{ - A_{2v} }} \Gamma \left( {A_{2v} } \right)B_{3v}^{{ - \left( {A_{3v} + 2} \right)}} } } \Gamma \left( {A_{3v} + 2} \right) - \left\{ {\hat{\lambda }_{3} } \right\}^{2}$$

35 $$\rho \left( {\hat{p}_{1} } \right) = \frac{1}{{\Omega_{v} }}\sum\limits_{i = 0}^{n - u} {\sum\limits_{j = 0}^{i} {\sum\limits_{k = 0}^{j} {\left( { - 1} \right)^{i} } \left( \begin{gathered} n - u \hfill \\ \, i \hfill \\ \end{gathered} \right)\left( \begin{gathered} i \hfill \\ j \hfill \\ \end{gathered} \right)\left( \begin{gathered} j \hfill \\ k \hfill \\ \end{gathered} \right)B\left( {B_{0v} ,C_{0v} } \right)B\left( {A_{0v} + 2,B_{0v} + C_{0v} } \right)B_{1v}^{{ - A_{1v} }} \Gamma \left( {A_{1v} } \right)B_{2v}^{{ - A_{2v} }} \Gamma \left( {A_{2v} } \right)B_{3v}^{{ - A_{3v} }} } } \Gamma \left( {A_{3v} } \right) - \left\{ {\hat{p}_{1} } \right\}^{2}$$

36 $$\rho \left( {\hat{p}_{2} } \right) = \frac{1}{{\Omega_{v} }}\sum\limits_{i = 0}^{n - u} {\sum\limits_{j = 0}^{i} {\sum\limits_{k = 0}^{j} {\left( { - 1} \right)^{i} } \left( \begin{gathered} n - u \hfill \\ \, i \hfill \\ \end{gathered} \right)\left( \begin{gathered} i \hfill \\ j \hfill \\ \end{gathered} \right)\left( \begin{gathered} j \hfill \\ k \hfill \\ \end{gathered} \right)B\left( {A_{0v} ,C_{0v} } \right)B\left( {B_{0v} + 2,A_{0v} + C_{0v} } \right)B_{1v}^{{ - A_{1v} }} \Gamma \left( {A_{1v} } \right)B_{2v}^{{ - A_{2v} }} \Gamma \left( {A_{2v} } \right)B_{3v}^{{ - A_{3v} }} \Gamma \left( {A_{3v} } \right)} } - \left\{ {\hat{p}_{2} } \right\}^{2} ,$$

where $v = 1$, $v = 2$ and $v = 3$ for the UP, JP and IP, respectively.

Also, the BEs and PRs under other three loss functions can also be derived. For sake of shortness, we have not given these derived BEs and PRs but presented upon request to the corresponding author.

## Elicitation of hyperparameters

Elicitation is a process used to enumerate a person’s prior professional knowledge about some unidentified quantity of concern which can be used to improvement any values which we may have. In Bayesian analysis, specification and elicitation of hyperparameters of prior density is a common difficulty. For different statistical models, different procedures for specification of opinions to elicit hyperparameters of prior distribution have been established.

Aslam^22^ suggested different methods which are depend upon the prior predictive distribution (PPD). In his study, one method based on prior predictive probabilities (PPPs) for elicitation of hyperparameters is used. The rule of evaluation is to link PPD with professional’s evaluation of this distribution and to select hyperparameters which make evaluation agree narrowly with a part of family. So, subsequent the rules of probability the professional would be consistent in elicitation of the probabilities. A few conflicts may arise which are not significant. A function $\Phi \left( {\xi_{1} ,\xi_{2} } \right) = \mathop {\min }\limits_{{\xi_{1} , \, \xi_{2} }} \sum\limits_{z} {\left\{ {\frac{{p\left( z \right) - p_{0} \left( z \right)}}{p\left( z \right)}} \right\}^{2} }$ can be applied to elicit the hyperparameters $\xi_{1}$ and $\xi_{2}$, where $p_{0} \left( z \right)$ represent the elicited PPPs and $p\left( z \right)$ denote the PPPs considered by hyperparameters $\xi_{1}$ and $\xi_{2}$. For elicitation, the above equations are simplified numerically in Mathematica. A method depend upon PPPs is considered to elicit the hyperparameters of the IP In this study.

### Elicitation of hyperparameters

Using the IP $\pi_{3} \left( {{\varvec{\Theta}}} \right)$, the PPD is define as:

37 $$p\left( y \right) = \int_{0}^{1} {\int_{0}^{{1 - p_{2} }} {\int_{0}^{\infty } {\int_{0}^{\infty } {\int_{0}^{\infty } {f\left( {y\left| {{\varvec{\Theta}}} \right.} \right)\pi_{3} \left( {{\varvec{\Theta}}} \right)d\lambda_{1} d\lambda_{2} d\lambda_{3} dp_{1} dp_{2} } } } } }$$

After substitution and simplification, the PPD is obtained as:

38 $$p\left( y \right) = \left\{ {\frac{{aa_{1} b_{1}^{{a_{1} }} }}{{y\left( {b_{1} - \ln y} \right)^{{a_{1} + 1}} }} + \frac{{ba_{2} b_{2}^{{a_{2} }} }}{{y\left( {b_{2} - \ln y} \right)^{{a_{2} + 1}} }} + \frac{{ca_{3} b_{3}^{{a_{3} }} }}{{y\left( {b_{3} - \ln y} \right)^{{a_{3} + 1}} }}} \right\}\frac{1}{{\left( {a + b + c} \right)}}.$$

Using the above PPD (22), nine integrals based on limits of $$Y$$, i.e., $$0.05 \le y \le 0.15$$, $$0.15 \le y \le 0.25$$, $$0.25 \le y \le 0.35$$, $$0.35 \le y \le 0.45$$, $$0.45 \le y \le 0.55$$, $$0.55 \le y \le 0.65$$, $$0.65 \le y \le 0.75$$$$0.75 \le y \le 0.85$$ and $$0.85 \le y \le 0.95$$ are considered with associated predictive probabilities 0.08, 0.07, 0.06, 0.06, 0.065, 0.07, 0.08, 0.09 and 0.10, respectively. It is stating that predictive probabilities may have been taken from professional(s) as their belief related to likelihood of given intervals. Now, to elicit the hyperparameters, the above equations are solved numerically using Mathematica software. From the above methodology, the values of hyperparameters are $$a_{1} = 0.9379$$, $$b_{1} = 0.8332$$, $$a_{2} = 0.7530$$, $$b_{2} = 0.6344$$, $$a_{3} = 0.5335$$, $$b_{3} = 0.4339$$, $$a = 2.4950$$, $$b = 2.5060$$ and $$c = 2.0200$$.

### Monte Carlo simulation study

From the Bayes estimators’ expressions, it is clear that analytical assessments between BEs (using priors and loss functions) are not suitable. Therefore, the Monte Carlo simulation study is used to assess the presentation of BEs under various loss functions and priors. Moreover, the presentation of BEs has been checked under sample sizes and test termination time. We calculated the BEs and PRs of a 3-CMPD through a Monte Carlo simulation as:

1. From given 3-component mixture distribution, a sample consists of $n \, p_{1}$, $n \, p_{2}$ and $n\left( {1 - p_{1} - p_{2} } \right)$ values out of *n* values is taken randomly from $f_{1} \left( y \right)$,$f_{1} \left( y \right)$ and $f_{3} \left( y \right)$, respectively.
2. Select values which are larger than $t$ as the censored values. The selection of $t$ has been prepared in such a way that there is approximately 10% to 30% censoring rate in resultant data.
3. Find the simulated Bayes estimates and posterior risks as $\hat{\omega } = \frac{1}{500}\sum\limits_{i = 1}^{500} {\left( {\hat{\omega }_{i} } \right)}$ and $\rho \left( {\hat{\omega }} \right) = \frac{1}{500}\sum\limits_{i = 1}^{500} {\rho \left( {\hat{\omega }_{i} } \right)}$, where Bayes estimates $\hat{\omega }_{i}$ and posterior risks $\rho \left( {\hat{\omega }_{i} } \right)$ of a parameter say $\omega$ are determine assuming censored values by solving (21)-(30).
4. Repeat steps 1–3 for $n = 30,{ 5}0,{ 1}00$, $\left( {\lambda_{1} , \, \lambda_{2} , \, \lambda_{3} , \, p_{1} , \, p_{2} } \right) = \left( {0.4,{ 0}{\text{.3}},{ 0}{\text{.2}}, \, 0.5, \, 0.3} \right)$ and $t = 0.9, \, 0.6.$

The simulated results have been arranged in Tables 2, 3, 4, 5, 6, 7, 8, 9. From Tables 2, 3, 4, 5, 6, 7, 8, 9, it is revealed that the extent of under-estimation of all five parameters assuming priors under SELF, QLF, PLF and DLF is larger for smaller *n* as compared to larger *n* for fixed *t.* Assuming fixed *n*, a similar trend is observed for smaller *t* as compared to larger *t*. It is also observed that PRs had inverse relationship with *n*, i.e., PRS decreased by increasing $n$ (cf. Tables 2, 3, 4, 5, 6, 7, 8, 9). Also, it is noticed that PRs had inverse relationship with *t*, i.e., PRS increased by decreasing $t$ (cf. Table 2, 3, 4, 5, 6, 7, 8, 9).

**Table 2:** The BEs and PRs under SELF with parameters $\lambda_{1} = 0.4, \, \lambda_{2} = 0.3, \, \lambda_{3} = 0.2, \, p_{1} = 0.5, \, p_{2} = 0.3.$

$t$	$n$	Priors	Bes				
			$\hat{\lambda }_{1}$	$\hat{\lambda }_{2}$	$\hat{\lambda }_{3}$	$\hat{p}_{1}$	$\hat{p}_{2}$
00.9	30	UP	0.460571	0.371987	0.279892	0.483369	0.303464
		JP	0.421334	0.337086	0.238015	0.483630	0.303647
		IP	0.439631	0.349391	0.267096	0.470931	0.297692
	50	UP	0.428565	0.344971	0.244023	0.489830	0.302109
		JP	0.416763	0.317347	0.225465	0.489892	0.302432
		IP	0.424941	0.337344	0.236809	0.481912	0.298489
	100	UP	0.411619	0.317893	0.222464	0.490724	0.300301
		JP	0.409768	0.313031	0.209039	0.494604	0.300767
		IP	0.412008	0.310983	0.214546	0.487511	0.303163

$t$	$n$	Priors	PRs				
00.9	30	UP	0.014584	0.016074	0.014424	0.007565	0.006397
		JP	0.012990	0.014721	0.011995	0.007568	0.006406
		IP	0.010828	0.012329	0.009833	0.006742	0.005794
	50	UP	0.007471	0.008203	0.006217	0.004773	0.004025
		JP	0.007396	0.007380	0.005875	0.004775	0.004028
		IP	0.007195	0.006987	0.004932	0.004366	0.003749
	100	UP	0.003277	0.003147	0.002560	0.001767	0.001389
		JP	0.003164	0.003053	0.002322	0.002111	0.001964
		IP	0.002965	0.002585	0.002241	0.001812	0.000512

**Table 3:** The BEs and PRs under QLF with parameters $\lambda_{1} = 0.4, \, \lambda_{2} = 0.3, \, \lambda_{3} = 0.2, \, p_{1} = 0.5, \, p_{2} = 0.3.$

$t$	$n$	Priors	BEs				
			$\hat{\lambda }_{1}$	$\hat{\lambda }_{2}$	$\hat{\lambda }_{3}$	$\hat{p}_{1}$	$\hat{p}_{2}$
00.9	30	UP	0.409332	0.296448	0.205024	0.448644	0.256064
		JP	0.374016	0.260241	0.158113	0.448952	0.257166
		IP	0.37478	0.280769	0.162495	0.414561	0.281108
	50	UP	0.404965	0.298157	0.196683	0.469611	0.273964
		JP	0.375158	0.277423	0.177027	0.469289	0.273502
		IP	0.389487	0.284741	0.195654	0.460878	0.289203
	100	UP	0.397235	0.301619	0.199885	0.485041	0.286571
		JP	0.389547	0.286446	0.187141	0.481864	0.292576
		IP	0.394696	0.29089	0.197311	0.460970	0.292947

$t$	$n$	Priors	PRs				
00.9	30	UP	0.068407	0.114832	0.169667	0.038115	0.085381
		JP	0.073221	0.12879	0.205113	0.038198	0.085509
		IP	0.064485	0.105065	0.184883	0.035298	0.071540
	50	UP	0.040769	0.068318	0.102349	0.021992	0.050101
		JP	0.042589	0.073490	0.113484	0.022016	0.050244
		IP	0.039444	0.065186	0.097374	0.021095	0.045039
	100	UP	0.012794	0.030941	0.049970	0.009186	0.024403
		JP	0.014569	0.077602	0.070874	0.014523	0.061045
		IP	0.012888	0.029994	0.030352	0.003537	0.019648

**Table 4:** The BEs and PRs under PLF with parameters $\lambda_{1} = 0.4, \, \lambda_{2} = 0.3, \, \lambda_{3} = 0.2, \, p_{1} = 0.5, \, p_{2} = 0.3.$

$t$	$n$	Priors	BEs				
			$\hat{\lambda }_{1}$	$\hat{\lambda }_{2}$	$\hat{\lambda }_{3}$	$\hat{p}_{1}$	$\hat{p}_{2}$
00.9	30	UP	0.473699	0.392311	0.305913	0.490801	0.313692
		JP	0.438033	0.363620	0.260598	0.491943	0.313734
		IP	0.454581	0.374621	0.244704	0.478091	0.309406
	50	UP	0.440035	0.357303	0.252612	0.494011	0.309304
		JP	0.422166	0.334364	0.229331	0.494023	0.309486
		IP	0.431558	0.351277	0.238926	0.48492	0.307309
	100	UP	0.419892	0.325579	0.223074	0.497226	0.304248
		JP	0.412053	0.314160	0.219033	0.498013	0.304786
		IP	0.407296	0.318637	0.22410	0.487496	0.297906

$t$	$n$	Priors	PRs				
00.9	30	UP	0.028845	0.038201	0.040083	0.015539	0.020761
		JP	0.028336	0.037192	0.039420	0.015563	0.020797
		IP	0.027668	0.035907	0.038064	0.014211	0.018355
	50	UP	0.016763	0.021748	0.021856	0.009720	0.013170
		JP	0.016717	0.021648	0.021716	0.009723	0.013189
		IP	0.016634	0.020992	0.021519	0.009087	0.012122
	100	UP	0.007254	0.011063	0.009877	0.005781	0.006787
		JP	0.007116	0.011043	0.009716	0.005793	0.006799
		IP	0.006623	0.009812	0.010062	0.002167	0.002596

**Table 5:** The BEs and PRs under DLF with parameters $\lambda_{1} = 0.4, \, \lambda_{2} = 0.3, \, \lambda_{3} = 0.2, \, p_{1} = 0.5, \, p_{2} = 0.3.$

$t$	$n$	Priors	BEs				
			$\hat{\lambda }_{1}$	$\hat{\lambda }_{2}$	$\hat{\lambda }_{3}$	$\hat{p}_{1}$	$\hat{p}_{2}$
00.9	30	UP	0.475799	0.408498	0.328774	0.497876	0.324916
		JP	0.453221	0.379994	0.282826	0.498166	0.325052
		IP	0.479645	0.388532	0.485623	0.481371	0.320722
	50	UP	0.447385	0.362848	0.268714	0.498202	0.315081
		JP	0.431255	0.345394	0.250602	0.499222	0.316196
		IP	0.443268	0.35825	0.249059	0.486872	0.31360
	100	UP	0.423969	0.309154	0.232522	0.499439	0.304887
		JP	0.408204	0.308525	0.222283	0.499570	0.307329
		IP	0.46566	0.339404	0.220779	0.487407	0.303426

$t$	$n$	Priors	PRs				
00.9	30	UP	0.059920	0.092535	0.126891	0.031455	0.065101
		JP	0.063718	0.101989	0.145478	0.031471	0.065119
		IP	0.056857	0.086621	0.118705	0.027287	0.056657
	50	UP	0.037738	0.059984	0.084845	0.019510	0.042297
		JP	0.039147	0.063918	0.092344	0.029497	0.042385
		IP	0.036388	0.046964	0.081050	0.018664	0.038305
	100	UP	0.023765	0.029997	0.039085	0.011914	0.015394
		JP	0.024099	0.035941	0.042387	0.019640	0.035477
		IP	0.021539	0.026378	0.026626	0.009967	0.014975

**Table 6:** The BEs and PRs under SELF with parameters $\lambda_{1} = 0.4, \, \lambda_{2} = 0.3, \, \lambda_{3} = 0.2, \, p_{1} = 0.5, \, p_{2} = 0.3.$

$t$	$n$	Priors	BEs				
			$\hat{\lambda }_{1}$	$\hat{\lambda }_{2}$	$\hat{\lambda }_{3}$	$\hat{p}_{1}$	$\hat{p}_{2}$
00.6	30	UP	0.502471	0.442411	0.405401	0.452371	0.311201
		JP	0.457120	0.409812	0.359421	0.460127	0.310249
		IP	0.468013	0.418756	0.365487	0.447627	0.314527
	50	UP	0.470024	0.390246	0.332946	0.467520	0.309874
		JP	0.432190	0.356914	0.304978	0.469985	0.309988
		IP	0.439901	0.359983	0.315912	0.450714	0.290121
	100	UP	0.446031	0.345217	0.271345	0.474198	0.305891
		JP	0.420101	0.338241	0.248127	0.472561	0.306467
		IP	0.425713	0.334251	0.259467	0.463786	0.293456

$t$	$n$	Priors	PRs				
00.6	30		0.234102	0.270027	0.269914	0.098421	0.089452
			0.230075	0.246321	0.224612	0.100294	0.092146
			0.194201	0.204672	0.17981	0.075614	0.069845
	50		0.169821	0.182791	0.154681	0.057842	0.042374
			0.156789	0.163087	0.132472	0.061247	0.042987
			0.110781	0.119897	0.104894	0.039872	0.030214
	100		0.086794	0.089814	0.067841	0.030918	0.021814
			0.079012	0.071237	0.032789	0.032179	0.022935
			0.024509	0.026127	0.020914	0.018974	0.010594

**Table 7:** The BEs and PRs under QLF with parameters $\lambda_{1} = 0.4, \, \lambda_{2} = 0.3, \, \lambda_{3} = 0.2, \, p_{1} = 0.5, \, p_{2} = 0.3.$

$t$	$n$	Priors	BEs				
			$\hat{\lambda }_{1}$	$\hat{\lambda }_{2}$	$\hat{\lambda }_{3}$	$\hat{p}_{1}$	$\hat{p}_{2}$
00.6	30	UP	0.415721	0.252012	0.155527	0.412341	0.231452
		JP	0.437801	0.244725	0.148792	0.415782	0.239854
		IP	0.438952	0.250868	0.150901	0.405271	0.256971
	50	UP	0.410216	0.268754	0.170264	0.438427	0.246923
		JP	0.368754	0.250011	0.165734	0.431798	0.250314
		IP	0.371548	0.257467	0.170012	0.420122	0.260341
	100	UP	0.398754	0.287906	0.185954	0.462346	0.268917
		JP	0.370241	0.275914	0.176458	0.469867	0.269898
		IP	0.379985	0.280347	0.180647	0.450012	0.278142

$t$	$n$	Priors	PRs				
00.6	30	UP	0.389534	0.47132	0.52641	0.152346	0.268674
		JP	0.412567	0.511230	0.665234	0.167714	0.280122
		IP	0.365491	0.442657	0.547889	0.123645	0.219850
	50	UP	0.302145	0.387564	0.402651	0.112340	0.185501
		JP	0.324598	0.425661	0.445620	0.123324	0.193325
		IP	0.268746	0.356001	0.378991	0.109875	0.166887
	100	UP	0.187564	0.275694	0.359870	0.075688	0.1234560
		JP	0.200344	0.299810	0.376900	0.098860	0.168985
		IP	0.142354	0.246010	0.293312	0.046772	0.102360

**Table 8:** The BEs and PRs under PLF with parameters $\lambda_{1} = 0.4, \, \lambda_{2} = 0.3, \, \lambda_{3} = 0.2, \, p_{1} = 0.5, \, p_{2} = 0.3.$

$t$	$n$	Priors	BEs				
			$\hat{\lambda }_{1}$	$\hat{\lambda }_{2}$	$\hat{\lambda }_{3}$	$\hat{p}_{1}$	$\hat{p}_{2}$
00.6	30	UP	0.535624	0.475234	0.440038	0.426920	0.333670
		JP	0.469201	0.442113	0.411126	0.429875	0.340140
		IP	0.475001	0.449919	0.420031	0.402210	0.330118
	50	UP	0.495002	0.429570	0.369986	0.440028	0.324542
		JP	0.4552477	0.403319	0.336792	0.446988	0.329987
		IP	0.463200	0.420301	0.343001	0.432100	0.321003
	100	UP	0.4564233	0.389906	0.276901	0.463651	0.318224
		JP	0.446681	0.355620	0.269841	0.469795	0.320345
		IP	0.440021	0.360028	0.274005	0.453327	0.311455

$t$	$n$	Priors	PRs				
00.6	30	UP	0.385501	0.451123	0.506724	0.125470	0.183321
		JP	0.374562	0.435670	0.467705	0.135432	0.170122
		IP	0.347701	0.394551	0.412200	0.113367	0.143774
	50	UP	0.225432	0.317739	0.386672	0.080443	0.125990
		JP	0.220101	0.293301	0.366011	0.089964	0.129987
		IP	0.199920	0.254332	0.304441	0.067300	0.106673
	100	UP	0.143544	0.226401	0.244312	0.055420	0.080021
		JP	0.136610	0.217943	0.224577	0.056011	0.088709
		IP	0.105773	0.163374	0.229943	0.034661	0.053318

**Table 9:** The BEs and PRs under DLF with parameters $\lambda_{1} = 0.4, \, \lambda_{2} = 0.3, \, \lambda_{3} = 0.2, \, p_{1} = 0.5, \, p_{2} = 0.3.$

$t$	$n$	Priors	BEs				
			$\hat{\lambda }_{1}$	$\hat{\lambda }_{2}$	$\hat{\lambda }_{3}$	$\hat{p}_{1}$	$\hat{p}_{2}$
00.6	30	UP	0.614231	0.530024	0.483540	0.416600	0.382451
		JP	0.574220	0.493455	0.438801	0.424322	0.394551
		IP	0.601124	0.503371	0.546788	0.409701	0.380052
	50	UP	0.536788	0.450774	0.411580	0.436321	0.366014
		JP	0.510114	0.427113	0.404551	0.439975	0.374402
		IP	0.530047	0.429989	0.326741	0.417330	0.355001
	100	UP	0.473310	0.376775	0.320046	0.463007	0.330767
		JP	0.460124	0.366609	0.319344	0.463551	0.355771
		IP	0.493320	0.400311	0.315664	0.458771	0.328003

$t$	$n$	Priors	PRs				
00.6	30	UP	0.365771	0.453304	0.503378	0.167012	0.323752
		JP	0.402257	0.474830	0.558332	0.175506	0.340057
		IP	0.346681	0.417745	0.479010	0.130221	0.278700
	50	UP	0.277740	0.361421	0.426609	0.117452	0.217740
		JP	0.289918	0.368892	0.457200	0.139820	0.227327
		IP	0.256744	0.317054	0.374450	0.105881	0.175584
	100	UP	0.193221	0.256772	0.303054	0.084452	0.135421
		JP	0.207784	0.266681	0.337451	0.106671	0.158544
		IP	0.168406	0.214771	0.273341	0.053347	0.098557

In case of choosing an appropriate prior, it is observed that IP materializes as an efficient prior because of lesser related PR as compared to NIP for estimating all five parameters under both symmetric and asymmetric loss functions (cf. Tables 2, 3, 4, 5, 6, 7, 8, 9). Also, it is noticed that JP (UP) emerges as a greater efficient because of smaller related PR as compared to UP (JP) for estimating component (proportion) parameters using both SELF and PLF (cf. Tables 2 and 6 vs Tables 4 and 8). Moreover, the UP is more efficient prior as compared to the JP under QLF and DLF due to smaller PR. On the other hand, the presentation of SELF is better than remaining three loss functions for estimating all parameters (cf. Tables 2 and 6).

It is also noticed that selection of an appropriate prior and loss function does not depend *t*. It is worth mentioning that our prior or loss function selection criterion is a posterior risk, i.e., we consider a loss function or prior the best if it yields minimum posterior risk as compared to others.

## A real-life application

Here, the analysis of a lifetime data to explain the procedure for practical situations is presented. Gómez et al.^23^ stated a lifetime data on exhaustion fracture of Kevlar 373/epoxy with respect to fix pressure at 90% pressure level till all had expired. Gómez et al.^23^ showed that data ${\mathbf{x}}$ can be modeled with an exponential mixture model. However, the $y = \exp \left( { - x} \right)$ as a transformation of an exponentially distributed data $\left( {\mathbf{x}} \right)$ provides the power random variable and we can use the resulting data to describe the proposed Bayesian analysis. The lifetime data are divided into three groups of values with 26 values from 1st subpopulation, next 25 values from 2nd subpopulation, and the last 25 values from 3rd subpopulation. To use type-I censored samplings, we used the 3.4 as a censoring time and noted down the ${\varvec{x}}_{1} = x_{11} , \, ..., \, x_{{1u_{1} }}$, ${\varvec{x}}_{2} = x_{21} , \, ..., \, x_{{2u_{2} }}$ and ${\varvec{x}}_{3} = x_{31} , \, ..., \, x_{{3u_{3} }}$ failed values from subpopulations I, II and III, respectively. The remaining values, which were greater than 3.4, have been taken censored values from each subpopulation. At the end of test, we have the following numbers of failed values, $u_{1} = 22$, $u_{2} = 22$ and $u_{3} = 21$. The remaining $n - u = 11$ values were assumed censored values, whereas $u = 65$ were the uncensored values, such that $u = u_{1} + u_{2} + u_{3}$. The data have been summarized as below:

$$n = 76,\;u = 65,\;n - u = 11,\;u_{1} = 22,\;\sum\limits_{w = 1}^{{u_{1} }} {\ln \left( {\frac{1}{{y_{1w} }}} \right)} = \sum\limits_{w = 1}^{{u_{1} }} {x_{1w} } = 31.2771$$

$$u_{2} = 22,\;\;\sum\limits_{w = 1}^{{u_{2} }} {\ln \left( {\frac{1}{{y_{2w} }}} \right)} = \sum\limits_{w = 1}^{{u_{2} }} {x_{2w} } = 32.3513,\;\;u_{3} = 21,\;\;\sum\limits_{w = 1}^{{u_{3} }} {\ln \left( {\frac{1}{{y_{3w} }}} \right)} = \sum\limits_{w = i}^{{u_{3} }} {x_{3w} } = 30.1508.$$

Here $n - u = 11$, therefore we have 14.5% approximately censored data. BEs and PRs are given in following Table 10.

**Table 10:** The BEs and PRs using the real life data.

Loss function	Prior	BEs				
		$\hat{\lambda }_{1}$	$\hat{\lambda }_{2}$	$\hat{\lambda }_{3}$	$\hat{p}_{1}$	$\hat{p}_{2}$
SELF	UP	0.761517	0.737372	0.755910	0.338405	0.338013
	JP	0.731365	0.708214	0.724613	0.338424	0.338007
	IP	0.770563	0.745674	0.761989	0.340009	0.339795
QLF	UP	0.700854	0.678693	0.692959	0.318327	0.317936
	JP	0.670828	0.649641	0.661789	0.318343	0.317925
	IP	0.711381	0.68806	0.699831	0.32191	0.321699
PLF	UP	0.776576	0.751934	0.771529	0.343172	0.342781
	JP	0.746384	0.722740	0.740190	0.343193	0.342776
	IP	0.785259	0.759976	0.777414	0.344328	0.344113
DLF	UP	0.791932	0.766784	0.787471	0.348007	0.347616
	JP	0.761711	0.737564	0.756103	0.348029	0.347612
	IP	0.800235	0.774552	0.793152	0.348702	0.348486

		PRs				
SELF	UP	0.023162	0.021687	0.023858	0.003249	0.003245
	JP	0.022864	0.021534	0.023746	0.003250	0.003246
	IP	0.022194	0.020786	0.022818	0.002956	0.002953
QLF	UP	0.041448	0.041408	0.043413	0.031014	0.031051
	JP	0.043140	0.043109	0.045282	0.031019	0.031059
	IP	0.039902	0.040155	0.042487	0.0277038	0.027716
PLF	UP	0.030117	0.029124	0.031238	0.009535	0.009536
	JP	0.030037	0.029052	0.031155	0.009537	0.009538
	IP	0.0293917	0.0286036	0.0308504	0.0086376	0.00863644
DLF	UP	0.038406	0.038356	0.040079	0.027591	0.027625
	JP	0.039839	0.039794	0.041648	0.027596	0.027631
	IP	0.037079	0.037283	0.039289	0.024928	0.024940

It is noticed that from the results, given in Table 10, are appropriate with the results given in simulation study section. The presentation of the BEs using IP is shown better than NIP as a result of smaller associated PRs for estimating all parameters under the different symmetric and asymmetric loss functions. Also, the BEs assuming JP (UP) is observed more suitable prior than UP (JP) based on smaller PRs for estimating component (proportion) parameters under SELF and PLF (SELF, QLF, PLF and DLF). In addition, it is revealed that the SELF is preferable to PLF, QLF and DLF due to minimum PRs for estimating all parameters.

Further to see how well the 3-CMPD performs as compared to other existing 3-component mixture distributions, we take 3-component mixture of exponential distributions (3-CMED), 3-component mixture of Burr type-XII distributions (3-CMBD), 3-component mixture of Rayleigh distributions (3-CMRD), and 3-CMPD. The Akaike information criterion (AIC) and Bayesian information criterion (BIC) are used to check their relative performance using the life span of fatigue fracture data. The AIC and BIC precise the relative loss of evidence so the lesser values of AIC and BIC reveal the best distribution. The AIC and BIC can be determined as: ${\text{AIC}} = 2k - 2\ln (L)$ and ${\text{BIC}} = k\ln (n) - 2\ln (L)$, where, *L* = likelihood value of given data, *k* = number of parameters in distribution and *n* = number of observations in data.

**Table 11:** AIC and BIC for different mixture distributions.

Mixture distributions	AIC	BIC	P-value (KS)
3-CMED	438.4067	428.4067	0.7865
3-CMBD	495.3025	485.3025	0.6754
3-CMRD	1324.493	1314.493	0.5745
3-CMPD	130.8868	120.8868	0.8245

It is observed from the results, given in Table 11, our proposed mixture distribution provides the least values of AIC and BIC as compared to the other mixture distributions and fits the best on the life span of fatigue fracture data. Also, the p-value of Kolmogorov-Smirnov (KS) test also indicates the proposed model fits better than the rest models.

## Conclusion and recommendation

In this article, a 3-CMPD using type-I right censored sample was developed to model lifetime mixture data using the Bayesian approach. Assuming the availability of IP and NIP under symmetric and asymmetric loss functions, the algebraic expressions of the BEs and PRs have also been presented. To assess the relative performance of BEs across different *n* with a fixed *t*, a comprehensive Monte Carlo simulation study has been performed. In addition to this, a real-life application has also been discussed to show the utility of the proposed methodology. From the results presented in the previous sections, we observed that as n increased, the BEs approached to their true value. To be more precise, smaller (larger) *n* results in larger (smaller) extent of under and/or over estimation at fixed value of *t*. We also noticed that the posterior risk decreased by increasing *n*. Finally, it is revealed that for a Bayesian analysis of 3-CMPD, the IP can be used to estimate component and proportion parameters under SELF. In future, the performance of the predictive distribution and predictive interval can be assessed. Also, other censoring schemes, like progressive and interval, can be used to develop mixture models in Bayesian framework.

## Data Availability

The datasets used and/or analyzed during the current study are available from the corresponding author on reasonable request.
